# Atypical Nelson Syndrome Following Right Partial and Left Total Nephrectomy With Incidental Bilateral Total Adrenalectomy of Renal Cell Carcinoma: A Chat Generative Pre-Trained Transformer (ChatGPT)-Assisted Case Report and Literature Review

**DOI:** 10.7759/cureus.36042

**Published:** 2023-03-12

**Authors:** Kyle Schuppe, Skyler Burke, Blake Cohoe, Kevin Chang, Raymond S Lance, Henry Mroch

**Affiliations:** 1 Internal Medicine, Washington State University Elson S. Floyd College of Medicine, Spokane, USA; 2 Urology, Washington State University Elson S. Floyd College of Medicine, Spokane, USA; 3 Nephrology, Washington State University Elson S. Floyd College of Medicine, Spokane, USA

**Keywords:** chatgpt improved case report, cutaneous hyperpigmentation, adrenocorticotropic hormone, von hippel-lindau, immune check point inhibitor adverse side effect, primary adrenal insufficiency, adrenalectomy, bilateral nephrectomy, renal cell carcinoma, nelson's syndrome

## Abstract

Nelson syndrome (NS) is a dangerous condition that can sometimes manifest after bilateral adrenalectomy (BA), typically in treating Cushing's disease. It is defined by the collection of systemic signs and symptoms that can arise in a state where there are chronically and massively elevated levels of adrenocorticotropic hormone (ACTH). Traditionally it may manifest from six months to 24 years following the loss of both adrenal glands, with the meantime of development being 15 years following BA. The diagnostic criteria are controversial, with historically many different methods being used, ranging from visual field defects and an enlarged pituitary corticotrophinoma to elevated plasma ACTH levels and skin hyperpigmentation. What remains consistent between criteria is that it is secondary to total BA, traditionally in treating refractory Cushing's disease. We describe here a rare case of a patient diagnosed with bilateral renal cell carcinoma (RCC) treated with right partial and left total nephrectomy, and incidental BA, presenting with the symptoms and signs of NS. Although NS classically presents following total BA for the treatment of Cushing disease, further research is required to look for etiologies of Nelson's-like pathology outside the context of Cushing's disease treatment, thereby necessitating a change to the traditional diagnostic criteria for the syndrome to identify cases that would otherwise go untreated. In addition, this case report's outlining, drafting, and conclusions were written in part by or with the support of Chat Generative Pre-Trained Transformer (ChatGPT), a large language transformer open-source artificial intelligence.

## Introduction

Nelson syndrome (NS) is a rare and potentially dangerous condition that can occur after bilateral adrenalectomy (BA) to treat Cushing's disease. It is characterized by a collection of signs and symptoms that can arise due to chronically elevated levels of adrenocorticotropin (ACTH) in the absence of functioning adrenal glands. The syndrome was first described by Dr. Don Nelson in 1958, who observed that some patients with Cushing's disease who underwent BA developed large pituitary tumors and clinical symptoms of hyperpigmentation and visual field defects [1]. The onset of NS can vary widely, with some patients developing symptoms within six months of surgery, while others may not experience symptoms for 24 years or more. The mean time of onset is around 15 years after BA. The diagnostic criteria for NS are controversial, with several methods used historically. These methods range from visual field defects and an enlarged pituitary corticotrophinoma to elevated plasma ACTH levels and skin hyperpigmentation [2-4]. Despite the controversy surrounding the diagnostic criteria, what remains consistent is that NS is secondary to total BA, traditionally in treating refractory Cushing's disease. The threshold plasma ACTH level varies among authors but can be anywhere from 200 pg/mL to 900pg/mL or more [2]. Here we present a rare case of a patient diagnosed with bilateral primary renal cell carcinoma (RCC) treated with a total right partial nephrectomy and left total nephrectomy, and incidental functional BA, that also presents with symptoms and signs indicative of NS. This case highlights the need for further research to investigate other incidences of Nelson's-like pathology outside the context of Cushing's disease treatment. This may necessitate re-evaluating the traditional diagnostic criteria for the syndrome and catching cases that would otherwise go untreated. In the new age of artificial intelligence (AI) in medicine, there remain questions about how AI should be used to augment and support physicians in medicine. Chat Generative Pre-Trained Transformer (ChatGPT), an open-source AI program, utilizes a language aggregation model to support human integration and offers a new tool to clinicians. ChatGPT functions as a query service where users can ask questions, complete written tasks, or explain complex topics [5]. An obvious use of ChatGPT is in the research, drafting, and editing of scientific manuscripts. This could save researchers time and offer new insights and perspectives. ChatGPT was utilized throughout the creation of this case report, and our experience with the program was documented.

## Case presentation

**Figure 1:**
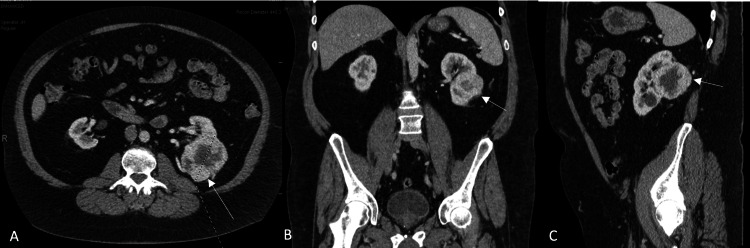
Left renal mass on contrast CT. The solid white arrows indicate the left renal mass in the transverse (1A), coronal (1B), and sagittal plane (1C).

**Figure 2:**
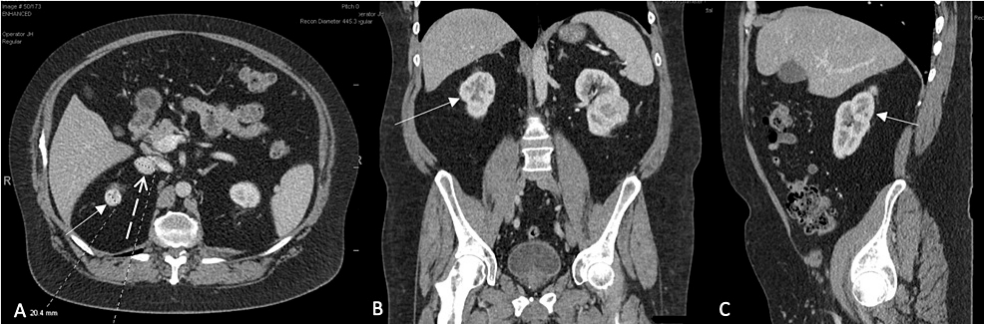
Right renal mass on contrast CT. The solid white arrows indicate the right renal mass in the transverse (2A), coronal (2B), and sagittal plane (2C), and the dashed arrow (2A) indicates the left adrenal mass.

A 57-year-old man presented with a two-week history of recurrent nausea, lack of appetite, fatigue, and inability to sleep, along with left lower quadrant pain and a feeling of fullness in his mid-abdomen. A colonoscopy was performed for suspected diverticulitis, and one 10 mm polyp was found in the terminal ileum with slight central umbilication and a 2 mm polyp in the descending colon. The rest of the examination was otherwise normal. The symptoms were attributed to irritable bowel syndrome, and the polyps were biopsied due to concerns for lymphoma. Biopsy results were inconclusive, and a CT abdomen-pelvis with contrast was performed for further characterization. Imaging revealed an 8.0 cm partially exophytic left posterior renal mass with central necrosis, consistent with RCC. The tumor extended centrally into the renal sinus, abutting Gerota's fascia posteriorly and crossing the axial renal midline (Figure 1). A 2.0 cm exophytic right upper pole renal mass located above the upper polar line was also found and was consistent with RCC (Figure 2). There was a concern for metastasis into the adrenals bilaterally due to the presence of adrenal nodules, with the left being 1.4 cm and the right being 2.8 cm.

**Figure 3:**
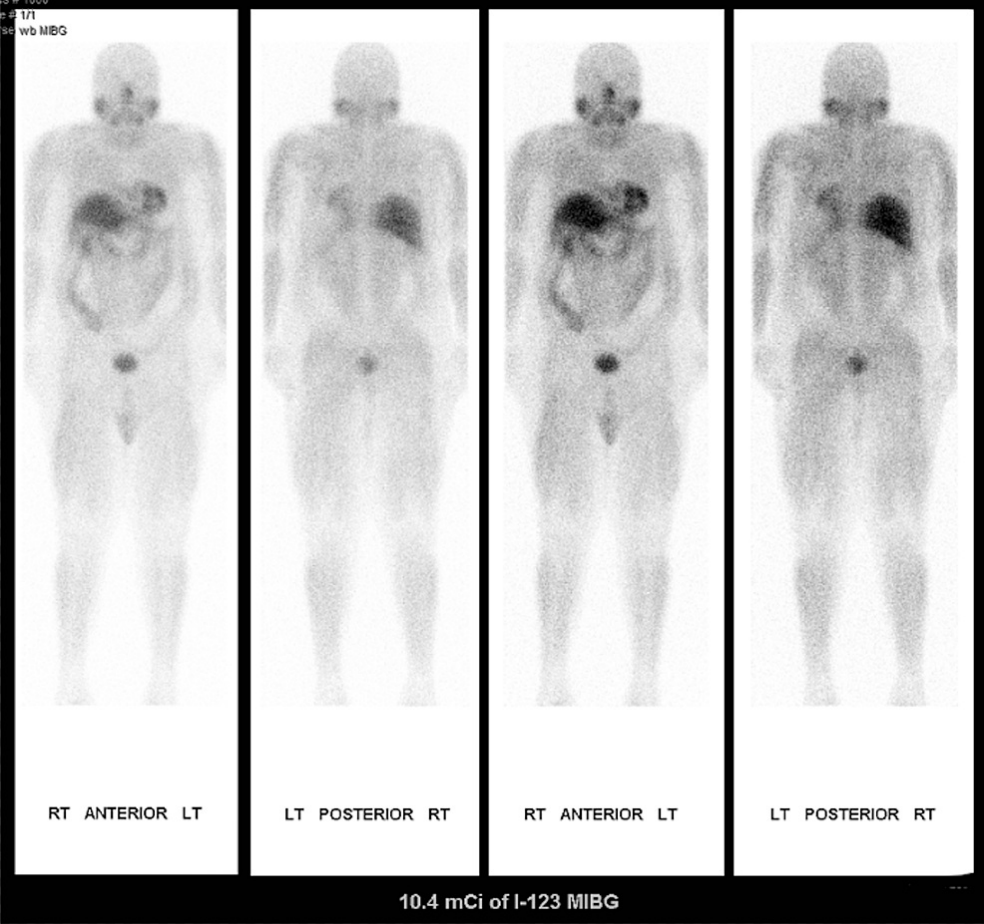
The meta-iodobenzylguanidine (MIBG) scan. Negative whole-body MIBG examination without abnormal radiotracer localization in the left adrenal gland nodule seen on comparison CT examination.

The patient was referred to a regional comprehensive cancer care center, where he was scheduled for a robotically assisted right partial nephrectomy and possible right adrenalectomy. In the interim, the patient was worked up by urology and found to have an elevated plasma norepinephrine of 1266 pg/ml [1,2,3], raising suspicion of pheochromocytoma. As a result, the adrenal biopsy was canceled, and the patient progressed to the planned resection of the adrenal mass. The patient was started on doxazosin 6 mg PO QD for the alpha blockade, and the surgery timeline was pushed forward. An evaluation of Von Hippel Lindau was negative, including an MRI and genetic testing. The operation was successfully completed. A 2.3 x 1.7 x 1.7 cm mass was removed from the right kidney, whose histologic analysis confirmed the diagnosis of renal clear cell carcinoma. Staging of the cancer was initially designated as pT1a pNX pM1 [4,5,6] with suspected metastasis into the ipsilateral adrenal gland. The tumor was staged as pT1a because it appeared to be confined to the kidney with no contiguous direct extension into the ipsilateral adrenal gland, despite apparent metastatic involvement. The right adrenal gland was not entirely removed, targeting only the mass. However, by that point, it had made up a sizable portion of the adrenal gland, and following mass resection and hemostasis, not much viable gland was left. Immunostaining of the right adrenal mass was inconclusive but seemed to be more indicative of a RCC metastasis rather than a pheochromocytoma. The final staging of the right tumor was pT4 pNX pM0. The operation had no complications, and the patient was discharged home after a couple of days of inpatient observation following surgery. Based on the staging and prognosis of his bilateral RCC with suspected metastases, this patient was enrolled in a pembrolizumab study receiving treatment every three weeks. His course was complicated by cytokine release syndrome [7-8]. A month and a half later, outpatient follow-up lab work revealed a further elevated plasma norepinephrine level of 1710 pg/mL. A month after this, his planned left nephrectomy was canceled in lieu of a recent syncopal episode with anosmia, acute kidney injury on chronic kidney disease (CKD) stage 3 with creatinine, and vision loss. Nephrology consultation attributed recent symptoms to possible adrenal insufficiency with renal hypoperfusion complicated by iatrogenic alpha blockade and catecholamine-induced volume contraction. Suspicion for a left-sided pheochromocytoma was high. Despite this, a meta-iodobenzylguanidine (MIBG) scan and metanephrine analysis were negative for pheochromocytoma (Figure 3). The patient markedly improved following excess antihypertensive removal and rehydration.

**Figure 4:**
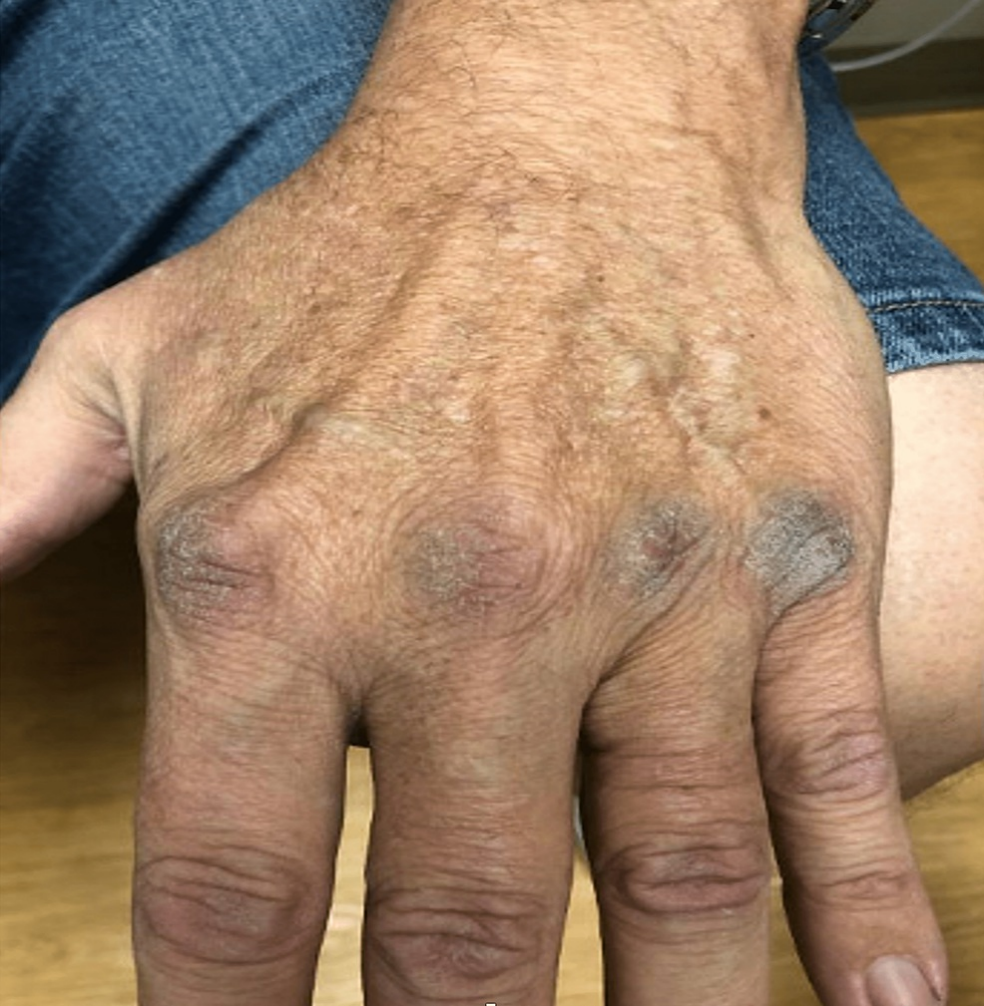
Hand hyperpigmentation. The dorsal hand shows a darkening of the knuckles and can be compared to the posterior leg.

**Figure 5:**
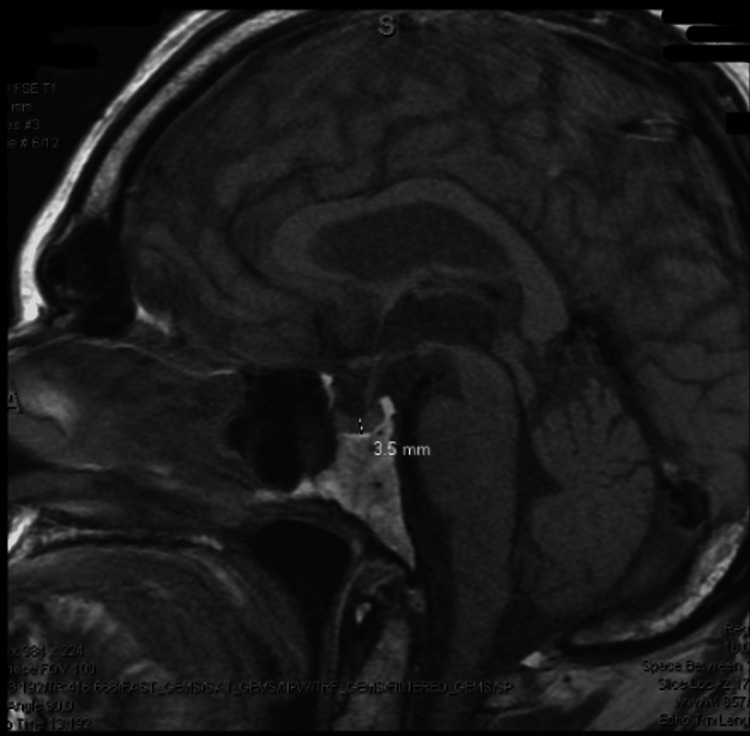
MRI of brain with contrast. The MRI shows the unremarkable appearance of the sella and pituitary gland, which measures approximately 3.5 mm in the cranial and caudal dimensions.

Two weeks later, the patient underwent a laparoscopic left radical nephrectomy and adrenalectomy. Final histopathology showed clear cell renal carcinoma without adrenal extension and no significant abnormalities in the ipsilateral adrenal gland, staged at pT1b pNX pM0 pR0 [9,10]. Due to final pathology reports not indicating pheochromocytoma in either adrenal gland, the prior elevations of plasma norepinephrine were attributed to the patient's use of venlafaxine for depression. Due to the functional BA, the patient now had adrenal insufficiency. During his hospitalization, the patient received IV hydrocortisone 50 mg three times per day and oral fludrocortisone 0.1 mg daily. He was hospitalized for seven days. The patient recovered well with intermittent nausea, pain, and an increase in creatinine which did return to baseline. At discharge, the patient continued 137 mcg of levothyroxine for his hypothyroidism and started on oral hydrocortisone 25 mg daily and fludrocortisone 0.1 mg daily. Given decreased renal mass, the patient was counseled on the importance of nephrology follow-up. The patient was also referred to endocrinology for continued management of his adrenal insufficiency. A few days after discharge, the patient followed up with endocrinology. The endocrinologist determined that the patient was taking adequate doses of hydrocortisone and fludrocortisone. The patient was educated on the possible side effects of steroid overdose and on increasing his hydrocortisone to 2-3 daily doses for 3-5 days in case of illness. The patient was also given injectable steroids if unable to tolerate oral medication. The patient was scheduled to return in one month. Shortly after the appointment, the patient fell ill, complaining of diarrhea. He was advised to increase his dose of hydrocortisone for 3-5 days, hold and subsequently decrease the dose of levothyroxine from 137 mcg to 112 mcg daily. At the follow-up one month later, the patient reported he had been feeling unwell for one week. He was experiencing back pain, generalized weakness, orthostatic hypotension, and decreased urinary output [11,12]. He continued to take his medicines correctly, not missing any doses, and kept his pills down with no nausea, vomiting, or diarrhea. The increased hydrocortisone dose was reported to be helpful. Two months later, the patient presented with weight gain and fluid retention. His hydrocortisone dose was decreased to 20 mg daily, and the patient was advised to decrease the fludrocortisone dose to half of a 0.1 mg tablet daily should swelling reoccur. On subsequent visits, the patient's hydrocortisone replacement was considered adequate, and the patient's adrenal insufficiency remained stable. Two years later, the patient's labs were notable for elevated ACTH at 796 pg/mL, and he was noted to clinically have atypical NS. Eight months after the initial finding of elevated ACTH levels, the patient presented with hyperpigmentation on both hands, darkening on the knuckles and palmar creases, and elevated ACTH at 696 pg/mL (Figure 4). Due to the patient's low BP and no weight loss, the patient's adrenal replacement regimen was left unchanged. In that interim, the patient had been compliant with his adrenal replacement, and his condition was well controlled. One month after the hyperpigmentation was noted, the patient underwent a pituitary MRI (Figure 5). The results of the imaging were unremarkable for any pituitary tumor. Per the endocrinologist's recommendations, the patient continued his hydrocortisone dose of 20 mg daily and half of a fludrocortisone 0.1 mg tablet daily. The adrenal replacement dose was adequate as his symptoms of NS improved over the course of the year. Morning ACTH samples trended downward from 796 pg/mL to 446.0 pg/mL, and the hyperpigmentation on his hands continued to resolve.

## Discussion

In this case, we discuss the endocrine manifestations of a patient who underwent a right partial and left total nephrectomy with functional BA for the treatment of RCC. RCC is the most common type of urogenital cancer with a mortality rate of 30-40% and is seen almost two-fold more commonly in men than women. In the United States, RCC accounts for an estimated 80,000 new cases and 14,000 deaths annually [6,7]. The incidence of lower-stage disease and overall survival has increased thanks to the incidental early discovery of RCC on imaging, as was the case in this patient. Genetic predisposition is related to a family history of RCC, but hereditary genetic conditions can also play a role [8,9]. One significant example is Von Hippel-Lindau disease (VHL), caused by a mutation in a tumor suppressor gene on chromosome 3. VHL often presents with neoplasia like pheochromocytoma, retinal angiomas, and hemangioblastoma [1,2,9,10]. While negative for genetic testing, given that this patient was initially suspected of having a pheochromocytoma, it was appropriate to evaluate him for VHL in this case. Like many malignancies, RCC has known risk factors contributing to oxidative DNA damage or loss of regulatory control, such as tumor suppression in VHL. Thus it follows that established risk factors for RCC, including smoking, obesity, hypertension, and both chronic and polycystic kidney disease, are most frequently associated with the development of RCC [7,10,11]. Although this patient did not smoke, he had hypertension, obesity, and CKD, which all likely played a role in his development of RCC [10,11]. Thus, it follows that preventative care should be the mainstay treatment before developing RCC. Patients should be counseled on increased dietary intake of fruits and vegetables, smoking cessation, weight loss, and adequate blood pressure control [11].

Secondary to lifestyle changes described for the prevention of RCC, treatment considerations for RCC depend on pathological and clinical staging [11]. Incidental or intentional characterization of lesions is defined by complexity, size, and enhancement using standardized guidelines such as those from the American College of Radiology [12]. If imaging is inconclusive, an ultrasound-guided biopsy may be indicated [13 ]. For smaller tumors with limited complexity on imaging and without metastasis or regional lymph node involvement, partial nephrectomy is associated with comparable outcomes compared to total nephrectomy [14]. A minimally invasive alternative to surgery for smaller non-complex tumors is interventional radiological radiofrequency ablation [15,16]. For more advanced RCC, chemotherapy treatment options can include tyrosine kinase inhibitors for growth factor receptors like sorafenib and sunitinib [17]. Anti-VEGF monoclonal antibody therapies such as bevacizumab have also shown benefits in the treatment of advanced RCC [18]. Newer immune checkpoint inhibitors such as nivolumab and ipilimumab can be effective in otherwise treatment-resistant metastatic disease. The patient described here underwent pembrolizumab therapy, but these therapies are not without adverse effects, such as cytokine release syndrome, which this patient experienced [19]. Bilateral RCC is rare, making up less than 5% of all RCC cases, and is predominantly seen in patients with known hereditary genetic conditions such as VHL, which this patient was known not to have [20,21]. It is thus exceedingly unusual that this patient was found to have bilateral RCC on incidental imaging and confirmed by post-operative biopsy following his total left nephrectomy and right partial nephrectomy. Bilateral adrenal involvement with RCC metastasis is also exceedingly rare, with one study estimating an incidence of ipsilateral and contralateral metastasis to be 3-5% and 0.7%, respectively [22-19]. Li Y et al. found that bilateral metastasis can be seen after nephrectomy to treat RCC [22]. Given the rarity of this finding, it explains why the patient was suspected of having a pheochromocytoma rather than bilateral adrenal metastasis [23, 24]. His MIBG and metanephrine levels were not indicative of a pheochromocytoma, and the biopsy of the right adrenal gland suggested RCC over a pheochromocytoma but was not definitive. The diagnostic approach to evaluating a suspected adrenal mass is not without challenges. Options include ultrasound, CT, or MRI-guided biopsy, but there is a risk of inducing hemorrhage, pneumothorax, or diaphragm perforation, especially in a patient with hypertension, suspected pheochromocytoma, and RCC [25]. Given these considerations, it was reasonable to undergo right partial and left total nephrectomy with incidental BA followed by a post-operative biopsy. Total BA is a rare surgery, as noted by Gagner et al., who found that only 6% of an audit of 1359 patients who underwent adrenalectomy had bilateral removal of the adrenal glands [26]. Some indications for BA include bilateral pheochromocytoma and ACTH-dependent hypercortisolism or Cushing's disease [26,27]. The post-BA complication of NS was first described in 1958 by Don H. Nelson, an endocrinology fellow with Howard Hughes Medical Institute and instructor at Harvard Medical School. Initially known as a postadrenalectomy syndrome, then later named after him, he described a patient who, three years after bilateral adrenalectomy for hyperadrenocorticism, was found to have an ACTH-secreting pituitary tumor [1,28]. Following BA, a rapid and dramatic cessation of endogenous cortisol production results in hypothalamic negative feedback-induced overproduction of corticotropin-releasing hormone (CTRH). This increased CTRH stimulates the pituitary gland to produce significant amounts of ACTH, resulting in pituitary adenoma [28]. Plasma fasting ACTH levels between 700 and 1000 pg/ml are usually predictive of NS, as was the case with the patient in this report (his peak ACTH was 796 pg/ml), but especially when combined with the presence of a pituitary adenoma on MRI [28,29]. The definition of NS, thus, is usually reliant on the presence of an ACTH-secreting pituitary adenoma secondary to BA; however, as in the case of the patient described here, a pituitary adenoma is not always present, which can confound the diagnosis of NS. Following BA, there is an estimated incidence of 0-47% (median 21%) of NS, which decreases with decreased BA rates [27,28]. Part of this variability may be due to disagreement on clinical definitions for NS, as discussed above. There is no consensus on how often MRI and visual disturbances should follow NS-induced pituitary adenomas or other mass effects are rare with appropriate medical management [27,30].

Treatment for NS depends on the presence of a pituitary adenoma, but, if present, patients should undergo microsurgery or endoscopic transsphenoidal resection. The resolution of symptoms occurs in roughly half of the cases and is complicated by post-operative hypopituitarism and diabetes insipidus in 30% and 20% of cases, respectively [28,31]. Another treatment alternative is radiation in the form of stereotactic radiosurgery or gamma knife, with similar outcomes in terms of hypopituitarism but better tumor control compared to surgery [28,32,33]. For atypical presentations of NS like this, patient surgery and radiation should not be undergone in the absence of a pituitary tumor. For all NS cases, including the one described above, all patients will require lifelong glucocorticoid replacement in the absence of endogenous cortisol, usually hydrocortisone 20 mg in the morning and 10 mg in the afternoon with mineralocorticoid supplementation [28,34]. This patient initially had under-corrected glucocorticoid replacement, developing iatrogenic Addison's disease with fatigue and orthostatic hypotension. Thus, he required an increase in his glucocorticoid supplementation, resulting in endocrinological control. Clinical manifestations of NS include hyperpigmentation of the skin, visual disturbances due to compression of the optic chiasm by pituitary adenomas, headache, generalized fatigue, and weakness. The most common manifestation of all is cutaneous hyperpigmentation which is clearly differentiated from other causes of hyperpigmentation. The linea alba, scrotum, and existing scars may all show increased discoloration [28]. The patient described here had substantial hyperpigmentation of both palmar and dorsal aspects of his hands and knuckles, intermittent fatigue, and weakness. Significantly, this patient had isolated hyperpigmentation as he did not have a pituitary adenoma on MRI. Thus this is an atypical presentation of NS, with the first manifestations being hyperpigmentation secondary to elevated ACTH. In complex cases like this, the assistance of a large language aggregation transformer like ChatGPT can be instrumental in identifying discussion points and clarifying language for the readers of medical literature [5,35]. The authors of this case report successfully used ChatGPT to edit and clarify the written components of this case report. For instance, we used it to edit the original conclusion and rewrite it for clarity which it did successfully (Appendix 1). However, it struggled to find citations with accuracy and occasionally would outright fabricate results when prompted to assist with finding relevant sources (Appendix 2). This finding is particularly challenging because many of the returned references sound plausible when considering the titles and journal names. That said, some of the references were legitimate and feasible options for authors looking to supplement a research paper. In summary, ChatGPT is a powerful research assistant if used properly. However, caution must be taken when using ChatGPT for identifying references or novel material for a research paper, and the results should always be confirmed.

## Conclusions

This case report discusses the presentation and management of a rare case of bilateral RCC with suspected bilateral adrenal gland RCC metastasis in a patient with no known hereditary genetic conditions such as VHL. Following surgical intervention for his RCC, the patient developed an atypical presentation of NS. Additionally, we also discuss our experience with ChatGPT in the making of this report. While this patient had elevated ACTH and cutaneous hyperpigmentation, there was no pituitary tumor that defined this condition. This patient's presentation suggests a need for further exploration of other incidences of Nelson's-like pathology. When using an AI chatbot trained to provide a response based on a prompt such as ChatGPT, the software excelled in editing and clarifying the language of this report. However, the software struggled with providing references and citations. While providing some legitimate sources of information, ChatGPT also created erroneous references with plausible titles, journal names, and authors.

## Figures and Tables

**Figure 6:**
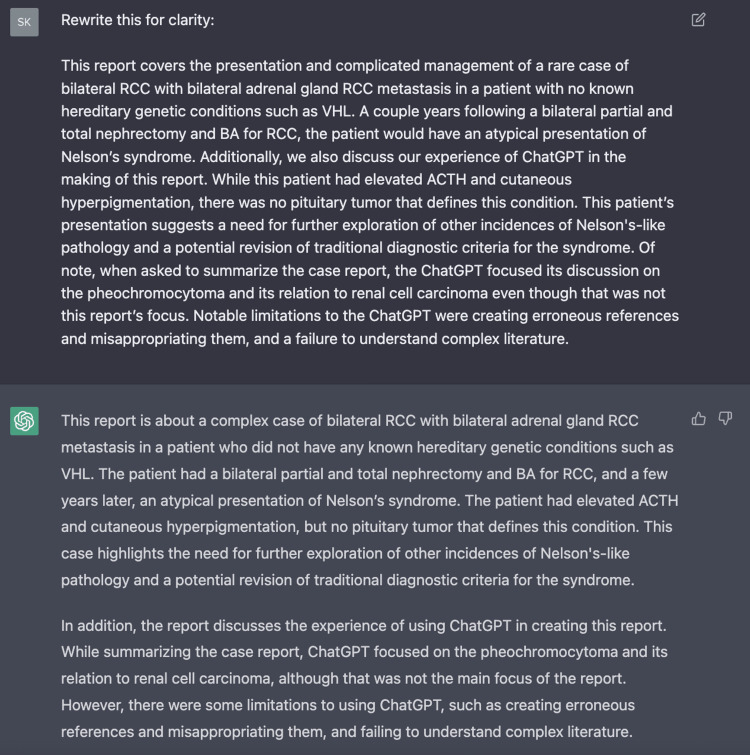
ChatGPT draft editing inquiry. ChatGPT's editing of an early draft of the conclusion. Notice how it edited portions of the conclusion into similar paragraphs but with better clarity.

**Figure 7:**
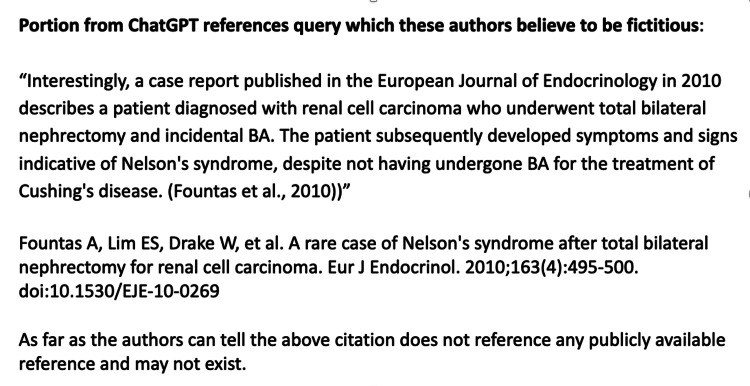
ChatGPT query response. A query response with a citation that these authors believe to be fabricated. A search on PubMed results in similar articles written by the first two authors separately and an appropriate sounding journal; however, the citation did not seem to exist.

## Appendices

Appendix 1 

Appendix 2 
